# Role of Optical Coherence Tomography on Corneal Surface Laser Ablation

**DOI:** 10.1155/2012/676740

**Published:** 2012-09-05

**Authors:** Bruna V. Ventura, Haroldo V. Moraes, Newton Kara-Junior, Marcony R. Santhiago

**Affiliations:** ^1^Ophthalmology Department, Altino Ventura Foundation, Recife, PE, Brazil; ^2^The Cole Eye Institute, The Cleveland Clinic Foundation, Cleveland, OH 44114, USA; ^3^Ophthalmology Department, Federal University of Rio de Janeiro, Rio de Janeiro, RJ, Brazil; ^4^Ophthalmology Department, Federal University of São Paulo, São Paulo, SP, Brazil

## Abstract

This paper focuses on reviewing the roles of optical coherence tomography (OCT) on corneal surface laser ablation procedures. OCT is an optical imaging modality that uses low-coherence interferometry to provide noninvasive cross-sectional imaging of tissue microstructure *in vivo.* There are two types of OCTs, each with transverse and axial spatial resolutions of a few micrometers: the time-domain and the fourier-domain OCTs. Both have been increasingly used by refractive surgeons and have specific advantages. Which of the current imaging instruments is a better choice depends on the specific application. In laser *in situ* keratomileusis (LASIK) and in excimer laser phototherapeutic keratectomy (PTK), OCT can be used to assess corneal characteristics and guide treatment decisions. OCT accurately measures central corneal thickness, evaluates the regularity of LASIK flaps, and quantifies flap and residual stromal bed thickness. When evaluating the ablation depth accuracy by subtracting preoperative from postoperative measurements, OCT pachymetry correlates well with laser ablation settings. In addition, OCT can be used to provide precise information on the morphology and depth of corneal pathologic abnormalities, such as corneal degenerations, dystrophies, and opacities, correlating with histopathologic findings.

## 1. Introduction

 Optical coherence tomography (OCT) is an optical imaging modality that performs noninvasive cross-sectional imaging of tissue microstructure *in vivo* [1]. It uses low-coherence interferometry to measure the delay and intensity of backscattered infrared light reflected from tissue structures [1]. The measurement is based on comparing the backscattering of tissue to light that travels a known reference path with a reference mirror [1]. To obtain an OCT image, the device creates a series of axial scans (A scans) and combines these scans to form a composite image [1]. OCT has transverse and axial spatial resolutions of a few micrometers and can detect reflected signals as small as 10^−10^ of the incident optical power [1].

Anterior segment OCT (AS-OCT) utilizes a superluminescent diode with a wavelength of approximately 1310 nm to produce high-resolution images of the anterior segment of the eye [2], achieving a 10- to 25-fold better resolution than high-frequency ultrasound imaging [3]. In refractive surgery, this device has been increasingly used in various clinical situations, such as central corneal thickness (CCT) measurement, preenhancement evaluation of flap and stromal bed thickness, and morphologic and depth assessment of corneal pathologic abnormalities. The purpose of this paper is to review the role of AS-OCT on corneal surface laser ablation procedures, focusing on the different devices available and their main clinical applications. 

## 2. AS-OCT Devices

Two AS-OCT devices that are commercially available are the Visante OCT (Carl Zeiss Meditec, Dublin, USA) and the Slit-lamp OCT (Heidelberg Engineering GmbH, Heidelberg, Germany). Both are time-domain OCTs; while producing their A-scans, the reference mirror subsequently changes in position to produce a reflectivity profile corresponding to depth. Therefore, the speed of these systems depends on the mechanical cycle time of the reference mirror driver [4], which limit the 3-dimensional (3-D) imaging of the ocular tissue. The Visante OCT has a transverse resolution of 60 *μ*m and an axial resolution of 18 *μ*m, while the Slit-lamp OCT has a transverse resolution of 75 *μ*m and an axial resolution of 25 *μ*m [5]. Both devices yield up to 2048 A scans per second [6].

Differently, fourier-domain (or spectral-domain) OCT has a stationary reference mirror and utilizes a spectrometer to detect the interference between the sample and reference reflections. Thus, image acquisition is faster than with time-domain OCTs [7], enabling 3-D imaging of the anterior segment [8]. The RTVue OCT (Optovue Inc., Fremont, USA) and the Cirrus OCT (Carl Zeiss Meditec, Dublin, USA) are fourier-domain OCTs that use light with a wavelength of approximately 830 nm. They were both initially developed to create images of the posterior segment. However, adjustments can be made to analyze the anterior segment. They have an axial resolution of 5 *μ*m and a transverse resolution of 15 *μ*m [7, 9]. The Casia SS-1000 OCT (Tomey Corporation, Nagoya, Japan) is a commercially available anterior segment fourier-domain OCT. As all AS-OCTs, it operates at a 1310-nm wavelength, having an axial and a transverse resolution of approximately 10 and 20 *μ*m, respectively [8, 10]. When comparing fourier-domain and time-domain OCT, the former achieves a higher resolution of smaller areas, allowing visualization of more tissue details, while the latter shows all anterior segment structures in a single image, which is sometimes essential in clinical practice [6, 11]. 

## 3. Role of OCT on Laser *In Situ *Keratomileusis (LASIK)

Laser *in situ* keratomileusis (LASIK) is an increasingly more common keratorefractive surgery worldwide. CCT is important for pretreatment screening and surgical planning [12]. AS-OCT can be used to evaluate this thickness. Previous studies have compared the OCT CCT measurement with the ultrasound pachymetry, which is the standard device used for measuring CCT. A strong correlation is seen between the CCT measurements with these two devices [12–15]. However, results vary regarding the difference in OCT and ultrasound pachymetry. While Kim et al. [13] have found no statistically significant difference between the measurements, other studies [12, 14–17] reported that the OCT pachymetry was systematically lower than the ultrasound pachymetry. It is unclear whether ultrasound or OCT measurements reflect more accurately the true corneal thickness. However, the repeatability of each device supports their application in CCT measurement although they should not be used interchangeably, and the CCT value should be interpreted in the context of the instrument applied [12, 14–17].

The preparation of the LASIK flap, which can be done with a microkeratome or a femtosecond laser, is the first critical step of the surgery [18]. In the past, the flap was evaluated exclusively by the central flap thickness [19]. This is still an important parameter for the safety of a LASIK procedure [20]. However, studies have shown the importance of analyzing the entire flap's morphology; although the basic differences in flap morphology regularity between femtosecond laser flaps and microkeratome flaps are in the periphery, the femtosecond laser flaps introduce significantly less lower-order and higher-order optical aberrations and lead to better visual acuity results [18, 21, 22].

The Visante OCT and a similar prototype have been used to assess the accuracy of the entire flap thickness and the flap morphology created by a femtosecond laser and a microkeratome [18, 23–25]. The repeatability analysis showed that the OCT flap thickness measurement was reliable for the entire extension of the flap [18, 23, 25]. The RTVue OCT also provides highly repeatable flap thickness measurements [9]. However, when comparing this device with the Visante OCT, the former is 13 times faster and produces an image with 3 times more resolution [11]. This resulted in a closer agreement in measurements between observers and between instruments and provided more consistent estimates of post-LASIK flap thickness [11]. Nevertheless, the RTVue OCT has the disadvantage of imaging a small tissue range, not scanning the angle recess or iris root [11]. Thus, each OCT design has distinct advantages, and which of the current imaging instruments is a better choice that depends on the application [4].

OCT devices best detect LASIK flaps in the corneal pericentral zone (2–5 mm in diameter), where the stromal bed signal is low, contrasting with the higher flap internal reflectivity and the flap interface peak [23]. Near the corneal vertex (<2 mm in diameter) the contrast is poor, because the interface reflections are overwhelming and both flap and bed internal reflectivities are high [23]. The higher resolution of the RTVue OCT enables flap measurements taken at 0.0 mm to be more reproducible than with the Visante system [11]. In myopic retreatments, the thinnest point is in the corneal center, making it the most important location for obtaining accurate thickness measurements for planning a subsequent surgery [11]. The increased flap internal reflectivity diminishes with time, but is present for at least 1 year after LASIK [24]. 

In addition to measuring flap thickness, OCT effectively assesses stromal bed thickness, which is an important measurement before an enhancement procedure [23, 24, 26–28]. A residual stromal bed thickness of less than 250 *μ*m post-LASIK is one of the main risk factors for keratectasia: a rare, but serious LASIK complication, characterized by progressive corneal distortion caused by weakening of corneal structure [20]. Although the current gold standard for assessing flap and stromal bed thickness is the intraoperative ultrasound subtraction pachymetry [24, 26], it has some disadvantages that led to the investigation of alternate methods for these measurements, such as the OCT. The intraoperative ultrasound flap pachymetry measurement is attained by subtracting the intraoperative stromal bed thickness from the preoperative corneal thickness, whereas the OCT determines this measurement by assessing the distance from the corneal surface to the flap interface [26]. In addition, there is room for OCT in identifying post-LASIK corneal ectasia in corneas with normal preoperative parameters, associated with a thicker than expected LASIK flap. Also, there may be other, as yet unidentified, risk factors for ectasia. Possible causes may include changes in preoperative corneal biomechanical properties or tomographic parameters and their correlation to alterations secondary to the cutting of a LASIK flap.

The Artemis 3-D very-high-frequency (VHF) digital ultrasound device (ArcScan Inc., Morrison, USA) is an option for evaluating the flap and residual stromal thickness. It has a LASIK flap thickness reproducibility of 1.14 *μ*m [29], which is better than the repeatability of the Visante OCT (4.5–17.6 *μ*m) [13, 24] and the RTVue system (4.19–4.9 *μ*m) [11]. Nevertheless, measurements with the Artemis device require direct ocular contact. Differently, OCT is a more practical technology because it is a noncontact method and has the benefit of providing high-resolution flap imaging over a wide area [12]. 

**Figure 1:**
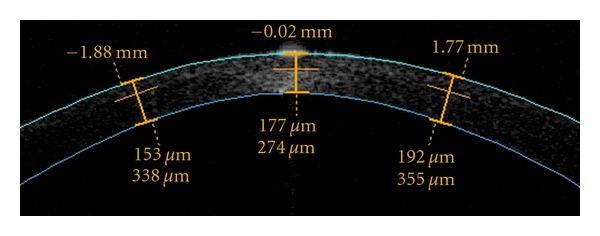
Optical coherence tomography showing a postoperative flap thickness of 177 *μ*m, producing a residual stromal bed of 274 *μ*m in the right eye. Data would help in case of an enhancement is needed.

In LASIK surgeries in which a femtosecond laser was used to create a flap, intraoperative ultrasound flap thickness measurements were similar to postoperative Visante OCT pachymetry [23, 24]. However, when a Hansatome microkeratome (Bausch & Lomb Surgical, Rochester, USA) was used, ultrasound measurements were thinner than OCT measurements [23, 24, 26]. The factors that explain this difference are not yet well understood. In one of the studies the authors [23] hypothesized that the flap hydration justified this finding. In this study, the OCT flap measurements were obtained 1 week after LASIK in all cases. The ultrasound stromal bed measurements were made 1 hour after the femtosecond laser cut and immediately after the microkeratome cut [23]. The authors believe that the real flap thickness was the one measured in the microkeratome group by the intraoperative ultrasound and that there was a hydration shift by the time the flap was measured 1 hour after its creation in the femtosecond group and 1 week postoperatively in both groups, resulting in thicker measurements [23]. However, this could not explain the findings of Murakami and Manche [24], as their OCT flap measurements were obtained 1 year after LASIK in all cases, when the edema had resolved [30].  Figure 1 is illustrative of flap thickness image with OCT.

A previous study [27] compared intraoperative ultrasound pachymetry with postoperative RTVue flap measurement in surgeries that used the Amadeus II microkeratome (Ziemer Ophthalmic Systems AG, Port, Switzerland) and Med-Logics blades (ML7090CLB; Med-Logics Inc., Laguna Hills, USA) to create all flaps. Both pachymetry devices attained equivalent measurements [27]. The OCT pachymetry was done 2 weeks after the procedure. This study did not include a group of patients in which the flap was created with a femtosecond laser. Possible explanations for these different findings when comparing to the aforementioned microkeratome results are the use of a fourier-domain OCT device in the later study [11], the different microkeratomes used [31], and the variability in postoperative time of OCT flap measurement [30]. Further studies are necessary to clarify these differences in OCT and ultrasound flap measurements to determine which is the most accurate method for flap thickness assessment. 

Nevertheless, when evaluating the ablation depth accuracy by subtracting preoperative from postoperative measurements, OCT pachymetry agreed better with the laser ablation settings than the ultrasound pachymetry [12]. The OCT provides an assurance before an enhancement procedure that sufficient stromal bed thickness is present, instead of detecting the insufficient bed thickness after lifting the flap and performing an ultrasound measurement during the procedure [12]. Furthermore, comparisons between the 1-week postoperative and the preenhancement OCT measurements could reveal any thickness change that may be associated with keratectasia [12], contraindicating an additional LASIK procedure. 

In the postoperative period, OCT enables identification of unexpected corneal changes, such as epithelial ingrowth [32]. OCT is also useful in assessing the recurrence of corneal dystrophy deposits after LASIK. The flap thickness and the location and the extent of the newly formed deposits are essential for determining the best treatment option [33]. 

## 4. Role of OCT in Excimer Laser Phototherapeutic Keratectomy (PTK)

**Figure 2:**
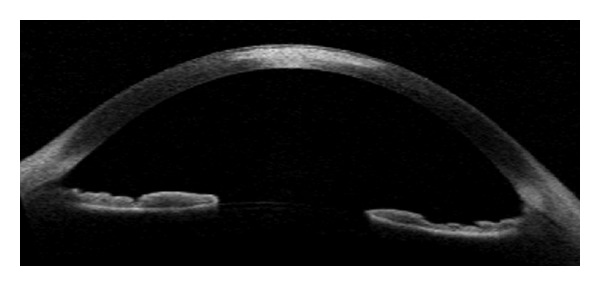
Optical coherence tomography showing corneal scar. The image is helpful in case photorefractive keratectomy is considered.

Excimer laser phototherapeutic keratectomy (PTK) is a treatment option for anterior corneal disorders that significantly degrade visual acuity. Three categories of conditions that are candidates for PTK are anterior corneal scars and opacities, elevated corneal lesions, and dystrophies of the epithelium, Bowman's membrane, and anterior corneal stroma. Although PTK can be used to treat disorders that affect the anterior one-third of the cornea, it is best suited for disorders in the anterior 10–20% of the corneal stroma. No more than one-third of the corneal thickness should be removed, and at least 250 *μ*m of stroma should remain after surgery [34]. Thus, an exam capable of producing precise corneal images independently of corneal transparency is necessary for screening possible candidates. OCT accurately maps corneal thickness in clear and opacified corneas, allowing the examiner to precisely map the depth of corneal abnormalities and plan the procedure [4, 33, 35, 36].  Figure 2 is illustrative of corneal scar image with OCT.

In cases with corneal scars and opacities, the Cirrus OCT has been shown to accurately measure corneal thickness and map the depth of these corneal abnormalities [32]. A previous paper compared the pachymetry of corneas with opacities measured with a high-speed anterior segment OCT prototype (Carl Zeiss Meditec, Dublin, USA), similar to the Visante OCT, an ultrasound pachymetry and an Orbscan II (Bausch & Lomb Inc., Rochester, USA) [37]. The OCT accurately measured corneal thickness and depth of corneal opacity, having similar results to the ultrasound pachymetry. Although the Orbscan II also provided pachymetric mapping, it significantly underestimated corneal thickness. Thus, OCT and ultrasound pachymetry are better imaging exams to assess corneal thickness in eyes with corneal opacities and to measure opacity depth for selecting and planning the appropriate treatment procedure [37].

Salzmann's nodular degeneration and keratoconus nodules are elevated corneal lesions [38, 39]. OCT can be used to assess the morphology of these nodules [40, 41]. Many of the established histopathologic findings can be seen using the Visante OCT *in vivo* [41]. In addition, OCT determines the nodule's depth, which will help define the best treatment strategy for each case [42]. 

Time-domain OCTs can also be used to assess eyes with corneal dystrophies and guide treatment decisions [6, 43]. Similarly to cases with Salzmann's nodules, in cases of corneal dystrophies the pattern of corneal deposits on the cross-sectional time-domain OCT scans correspond to that on histopathologic serial sections [36]. However, the higher resolution of fourier-domain OCTs allows visualization of even more details [6]. The Cirrus OCT can accurately map corneal dystrophies [32]. It has been shown that the RTVue device enables the visualization of corneal guttae on the posterior corneal surface of eyes with Fuchs corneal dystrophy, irregularity of Bowman layer in eyes with macular dystrophy, lattice lines in eyes with lattice corneal dystrophy, and morphology and depth of the deposits in eyes with granular corneal dystrophy type 2 [6, 44]. It also allows visualization of subepithelial fluid in eyes with bullous keratopathy [6]. 

Before introducing fourier-domain OCT, the corneal surface was ablated to some degree, and the remaining corneal opacity was examined using the slit lamp in the sitting position. The corneal ablation and slit lamp examination were repeated until vision-threatening deposits were removed [33]. Differently, some authors have shown that the actual required PTK ablation depth correlates well with the depth predicted by a fourier-domain OCT [45]. However, even though the fourier-domain OCT has a better resolution than the time-domain devices, in clinical practice it is sometimes essential to assess all anterior segment structures in a single image [6]. Thus, the choice of the device used in each case is based on the reason that led to the exam [4]. 

## 5. Conclusion

In the context of corneal surface laser ablation procedures, the time-domain and fourier-domain OCTs can be used. Both OCT designs have distinct advantages, and the better choice depends on the needs of each particular case. OCT can be used to accurately measure CCT, to evaluate the regularity of LASIK flaps, to quantify flap and residual stromal bed thickness, and to evaluate post-LASIK complications. Furthermore, it provides precise information on the corneal structure, and on the morphology and depth of corneal pathologic abnormalities, which help define the best treatment strategy for each patient.
